# Seroprevalence survey of SARS-CoV-2, community behaviors, and practices in Kansanshi and Kalumbila mining towns

**DOI:** 10.3389/fpubh.2023.1103133

**Published:** 2023-09-20

**Authors:** Temple Kahilu Mumba, Kylie Van Der Merwe, Mark Divall, Kelvin Mwangilwa, Nkomba Kayeyi

**Affiliations:** ^1^Health and Wellness, First Quantum Minerals Limited, Solwezi, Northwestern, Zambia; ^2^Zambia National Public Health Institute, Surveillance Disease and Intelligency Cluster, Lusaka, Zambia

**Keywords:** COVID-19, prevalence survey, cluster, vaccine uptake, community sensitization

## Abstract

**Background:**

Coronavirus disease 2019 (SARS-CoV-2) was declared a global pandemic by WHO after it spreads quickly around the world from its source city in Wuhan. Africa has some of the lowest documented SARS-CoV-2 incidences globally, with over 9 million confirmed cases as of December 2022. This may be due to efficient mitigation, outbreak response, or demographic traits. Surveillance capability may have suffered as nations changed funding, regulations, and testing plans. Therefore, this study was to document the prevalence of SARS-CoV-2, its characteristics, and the socio-economic characteristics in the two mining districts of Solwezi and Kalumbila of Zambia.

**Methods:**

Between 28 March and 26 April 2021, a cross-sectional cluster-sample survey of households in two mining districts of Zambia was conducted. Twenty standard enumeration areas (SEAs) were randomly selected in Kansanshi (17 SEA) and Kalumbila (3 SEA) from a total of 67 SEA that encompass the two mines. Members of households aged <5 years were not eligible to participate in the survey. All participants that consented to participate in the interview were also asked to consent to test for SARS-CoV-2 infection using a rapid diagnostic test (RDT), which tested for recent infection and past exposure to the virus (IgM and IgG, respectively).

**Result:**

Out of the total sample of 3,047 that were present for the interview, 622 of them agreed to test for COVID-19. Of the total that tested for SARS-CoV-2, 2.6% were IgM positive while 9.0% were IgG positive. Despite the above results, 1,586 participants that agreed to the interview indicated a low self-risk assessment of getting COVID-19 (46.5%) or someone (45.5%). On the public health measures, participants who did handwashing more than usual (65.0%), not hand sanitizing more than usual (69.0%), not disinfecting surfaces in their households than usual (87.5%), not avoiding drinking from bars or nightclubs (90.6%), and not wearing a mask when out in public places (71.1%). In the logistic multivariable model, participants with age 24 years and above (AOR = 2.94; 95% CI = 1.10, 7.81) and having experienced symptoms of SARS-CoV-2 (AOR = 2.60; 95% CI: 1.33, 5.05) had a significant effect on testing positive for SARS-CoV-2.

**Conclusion:**

Although the results showed that active COVID-19 prevalence in Solwezi and Kalumbila communities surrounding the two mines was low, exposure to infection was five times high. Government and mining firms should continue to sensitize the community members on the preventive measures of COVID-19 and continue with community testing so that all those positive but without symptoms can self-isolate and those with symptoms and sick can be admitted to the hospital.

## Background

SARS-CoV-2 was declared a global pandemic on 11th March 2020 by WHO. On 16th July 2023, the world recorded 767 million positives and 6.9 million deaths. During the same period, Zambia recorded 347,022 cases of COVID-19 with 4,062 deaths (1). The North-Western province was identified as one of the hotspots of the pandemic in Zambia, with 6,073 cases being recorded during the same period. Solwezi and Kalumbila districts contributed 3,076 cases and 854 cases, respectively, to the total number of cases reported in the North-Western province. Further SARS-CoV-2 effects differ significantly across various social, political, and geographic contexts (2).

Early COVID-19 forecasts suggested a sizable morbidity and mortality burden in Africa due to COVID-19. However, with over 9 million verified cases as of December 2022, Africa has some of the lowest known incidences of COVID-19 in the entire world (3). It is yet unknown, though, whether this is due to effective mitigation, outbreak response, or demographic characteristics. As nations altered their funding, national legislation, and testing strategies in response to the COVID-19 pandemic, surveillance capability may have decreased. Currently, Africa has far lower death rates than other regions, which is sometimes attributed, among other things, to the continent's youthful population (4), but in the majority of African countries, community transmission is currently accelerating (5). Although the epidemic is spreading most swiftly in South Africa, where there are the most cases, the bulk of COVID-19-related deaths in Africa have occurred in Egypt. Even if testing and case data in Africa are not as complete as those in other areas of the world, the pandemic's impact on economies and health systems cannot be denied. Aside from Ghana and Nigeria, other countries that have suffered greatly include Algeria, Morocco, and Morocco (6).

The virus was mainly spread between people during close contact, often via small droplets produced by coughing, sneezing, or talking (7). Other people got infected by touching contaminated surfaces and then touching their faces (8). To prevent COVID-19 transmission, the Zambian government, such as most governments around the world, instituted preventive measures such as masking up when in public, social distancing, maintaining personal hygiene by washing hands or hand sanitizing, closing high spreader places, and stopping high spreader events. Other public health measures instituted included the closure of schools and higher learning institutions, restriction of public gatherings, requiring all restaurants to operate only on take away and delivery basis, and closure of all bars, nightclubs, cinemas, gyms, casinos, banks, mines, government and private institutions, schools and universities both private/government, and all international flights to land and depart from Kenneth Kaunda International Airport in Lusaka (9). Zambia is a land-linked country with ten provinces, and all the provinces reported case of COVID-19 by 16th of July 2023. The North-Western province is in the North-Western part of Zambia and has two mining districts (Kalumbila and Solwezi). The measures were also implemented in the two mining districts of Solwezi and Kalumbila in North-Western province of Zambia. Furthermore, supportive interventions were implemented to mitigate the COVID-19 socio-economic and mental health shocks among the miners and their families.

As COVID-19 is a new disease, many knowledge gaps exist on the magnitude of spread among the community members, especially that most of them were asymptomatic (10), its virulence, and infective dosage (11). Questions also remain unanswered surrounding its symptom manifestation, which so far range from mild to severe, particularly, the role of subclinical infections in person-to-person transmission and if persons with subclinical presentation can transmit to other individuals (12). During interventions of public health measures, different health promotion mechanisms were used and it is important to document the most effective methods and if people were adhering to the preventive measures instituted by the government. Mental health effects of COVID-19 on the miners and theirs need to be documented.

A study conducted in July 2020 in Kabwe, Livingstone, Lusaka, Nakonde, Ndola, and Solwezi districts showed a high prevalence of acute SARS-CoV-2 infection in the community but a low prevalence of antibodies among participants, meaning there had been few previous infections (13). However, that study was not representative of the entire country as the six districts were purposefully selected. Additionally, among confirmed cases in Zambia, most have been reported from Lusaka District (Zambia National Public Health Institute, 2020) (13). However, the completed prevalence study from six districts indicated equivalent SARS-CoV-2 burden, suggesting a potential reporting bias resulting from more tests being performed in Lusaka District compared to elsewhere in Zambia. Prevalence surveys that measure the distribution of SARS-CoV-2 in the population are important tools to address this bias from uneven distribution of testing in Zambia, and SARS-CoV-2 prevalence survey will better help assess the extent and nature of a reporting bias (14).

While this is important for policy decision-making, nationally, health and wellness program had an interest in understanding disease transmission in its wider community in Solwezi and Kalumbila to determine the effectiveness of controls and demographic spread in the mining areas. This was to support the evidence for better workplace controls and understanding of behaviors and practices to better inform education and communication programs. Ultimately, First Quantum Minerals (FQM) supported the desire for zero harm for improving health and wellness in its communities. Therefore, this study aims to document the prevalence of COVID-19, its characteristics, and the socio-economic characteristics in the two districts of Solwezi and Kalumbila of Zambia. The benefit of the study was that knowing the determinants of the community burden of COVID-19 would help put up specific interventions aimed at improving service delivery eventually improving the management of COVID-19 in Zambia and future similar outbreaks.

## Methodology

### Study design

The COVID-19 seroprevalence survey was a cross-sectional study, and a similar method has been used elsewhere (10, 13, 15). In this study, 800 households with an estimated 3,045 persons in communities in the Solwezi and Kalumbila districts of North-Western province were involved.

#### Study areas and participant population

The COVID-19 prevalence survey included the following populations in Solwezi and Kalumbila districts: the miners and their dependents. The study participants were recruited through the household survey of communities surrounding the mines, with a population of 203,799 in the Solwezi district and 127,604 in the Kalumbila district. A household listing of selected standard enumeration areas (SEAs) was conducted, and 40 households per SEA were randomly selected. All persons in the selected SEA aged >5 years were eligible for the survey, which gave an estimated sample size of 3,200 persons.

#### Sampling design

A two-stage sampling methodology was used to recruit participants for the household survey.

The primary sampling unit (PSU) was the standard enumeration area (SEA) that was selected using probability proportional to size (PPS) of the SEAs. A total of 20 SEAs were randomly selected from the 2010 Census of Population and Housing sampling frame (16). The sampling frame in the targeted areas of Solwezi and Kalumbila districts has 61 SEA, and it details the estimated total number of households and the estimated population per SEA. Each SEA has a cartographical map that shows the boundaries, which do not overlap, and the main landmarks. In this study, Kamitaka township, Kimalamba, Trident town, Northern Resettlement, Kisasa, Kansanshi golf estate, and Kimasala were the communities considered for enumeration as they are areas where mine workers live with their dependents.

The sampling process began by stratifying areas around the two mines that are Kansanshi in the Solwezi district and Kalumbila in the Kalumbila district into SEAs. The SEAs were further stratified by rural–urban areas. Sampling weights for each SEA were calculated, taking into consideration that each SEA has a different sampling probability of selecting a household. In the second stage of sampling, we used systematic random selection to select a fixed number of 40 households in each selected SEA. Using the household listing software, the survey team listed all households in the selected SEA and randomly selected 40 households. A total of 800 residential households were selected for the survey. In each selected household, all eligible household members (aged ≥ 5 years) who gave consent were interviewed. The target sample was 3,200 persons for the survey.

##### Inclusion criteria

The following was used as the criteria for recruiting participants in the study: All participants living in the selected households in each selected SEA provide informed consent/assent to participate in the study and consent to drawing blood specimens and/or being swabbed.

##### Exclusion criteria

All participants who already tested for COVID-19 and had a positive result were excluded.

### Data collection methods

The field team comprised data collectors and phlebotomists. These data collectors and phlebotomists (blood collectors) were fluent in the local languages (Kaonde, Lunda, and Luvale) and understand the culture of the communities. They were trained in research ethics (17), data collection questionnaire implementation, biosafety, quality assurance, and correct use of PPE and worked under the supervision of a team leader. The phlebotomists were furthermore oriented on the best practice of finger prick and capillary tests. Data collectors and supervisors were trained on how to complete the questionnaire on the tablet. Additionally, the field teams were equipped with Infection Prevention and Control (IPC) and safety handling practices. Data quality was also controlled by close supervision, data cleaning and editing, and cross-checking of the completeness of the questionnaires. The questionnaire was pre-tested in similar settings which were not part of the study area, and the necessary modifications were made on some items of the questionnaire.

On completion of the questionnaire, the participant was referred to the phlebotomist for a finger prick to test for COVID-19 using RDT antigen per standard procedures defined by the manufacturer of the rapid detection kits (Onsite Rapid Test^®^) (18–20). The samples were analyzed following the manufacturer's instructions with the result recorded on a laboratory form and entered into the tablet. All tests were performed according to the manufacturer's instructions and approved survey standard operating procedures (SOPs). SOPs were adhered to during the entire survey period, including procedures for testing, biosafety, and waste disposal (18).

A standardized COVID-19 questionnaire was used to collect information on demographic data (age, sex, occupation); household socio-economic situation; knowledge attitudes and practices toward COVID-19; and comorbidities and healthcare seeking.

After consenting, a face-to-face interview was conducted in a local language with all eligible members of the household using a tablet device. The exceptions were children under 5 years who were not subjected to a questionnaire. The data manager checked for completeness of the COVID-19 questionnaire on the chosen electronic platform during and at the end of each day. These data were loaded on a database that links to the questionnaire.

#### Laboratory procedures

A standard finger prick blood sampling method was used to obtain a capillary blood sample from consenting participants. This was performed using universal protection and according to best practices (21). All blood samples were analyzed in the field, and the results were available within 15 min as SARS-CoV-2 rapid diagnostic tests were used. The result was recorded with one of the following outcomes if the participant was exposed to the virus and got infected or was negative (21). The results of the test were shared with the participant or the parent/legal guardian immediately. Privacy provision was ensured when the results were shared. With participants that tested positive for the RDT test whether IgG or IgM, we checked whether they are exhibiting any symptoms (9). For those with symptoms, a team was put on standby to go and test them using the PCR test, and if the PCR test became positive, then national guidelines were followed (22). All medical wastes were collected in biohazardous bags and incinerated at the ZEMA-approved incinerator at Kansanshi mine.

##### SARS-CoV-2 antibody testing

A rapid diagnostic test (RDT) was selected for the survey to address numerous logistical challenges (Onsite Rapid Test, CTK Biotech, USA). The test is reported to have a 97% sensitivity and 97.8% specificity when compared to PCR samples and the RDT test kits (23).

### Ethical review and data security

Ethics approval was granted for the study by the University of Zambia, Biomedical Research Ethics Committee (UNZABREC: REF. NO. 1660/2021). The data sets did not have participants' names but had an identification number, and hence, anonymity and confidentiality were guaranteed.

As previously described, survey data were entered directly onto a tablet during the interview. Data from the tablet were removed from the device when transferred to the central database. Data entry checks were put in place during the interview to prevent illogical data values. Interview data were password-protected on encrypted tablets and were stored in central databases on encrypted, password-protected computers at the study sites. All data and survey results were kept confidential and stored in a safe place. Paper data containing household, locator, and identifying data were kept separate from the interview data in a secure locked cabinet, in locked offices. The Public Health Coordinator at Zambia National Public Health Institute was responsible for making sure that all documents are properly secured. Hard copies of consent forms will be kept for 5 years beyond the end of the study and then destroyed. All ethics procedures were also followed using international ethical guidelines (17).

### Data processing

#### Data analysis

Data were cleaned containing age, sex, and occupation; household socio-economic situation; knowledge attitudes and practices toward COVID-19; and comorbidities and healthcare-seeking behaviors, with a total of 1, 586 participants. Prior to analysis, the data were weighted by primary sampling unit, stratum, and sample weight to ensure the survey's representativeness. Descriptive statistics were performed to observe the characteristics of the variables (numbers and percentages were reported as the variables were categorical). To account for the complex multistage sampling design and the clustered nature of the data, survey analysis approaches were used. Since the conditions for a chi-squared test were met, associations between categorical variables were evaluated using the chi-squared test of independence. The vce (cluster comp) syntax in the Stata version 15 command was used to take into consideration the complex multistage sampling design and the clustered nature of the data.

The primary statistical analysis used univariable and multivariable logistic regression to determine the factors that predicted SARS-CoV-2 infections among the mining community. Multiple logistic regression was used to select variables influencing SARS-CoV-2 using an investigator-led stepwise regression procedure. The final multiple regression model's variables were chosen by first running the multiple logistic regression command with all of the predictor variables and then removing one by one the predictor variables with the highest *p*-values from the model until only those predictor variables that best predicted the outcome remained in the model. Finally, based on Akaike's information criterion and Bayesian information criterion (AIC and BIC) for the competing models, the best-fit model was chosen. The model that had the lowest AIC and BIC values in comparison with other models was picked. The 95% confidence intervals (CIs) for the crude odds ratio (cOR) and adjusted odds ratio (aOR) were shown. A *p*-value of 0.05 was regarded as significant. STATA 15 (STATA Corp.) was used to analyze the data.

## Results

### Sample characteristics

#### Summary of the results

Out of 622 respondents, 9.0% tested positive for IgG and 2.6% for IgM, with more positive responses in the Solwezi district. The study examines respondents' knowledge levels and self-risk assessment of COVID-19 symptoms in Zambia. Most respondents believe that close contact with infected individuals can lead to infection, with only 2.3% having no knowledge of COVID-19 transmission. Most respondents assessed their self-risk as no chance (27.4%), very small chance (19.1%), and medium chance (27.1%), with no differences between Solwezi and Kalumbila districts. Respondents trusted information on COVID-19 from healthcare providers, with 56.2% trusting the Ministry of Health website. Participants with age 24 years and above as well as having experienced symptoms of SARS-CoV-2 had a significant effect on testing positive for SARS-CoV-2.

#### Distribution of sample

**Figure 1:**
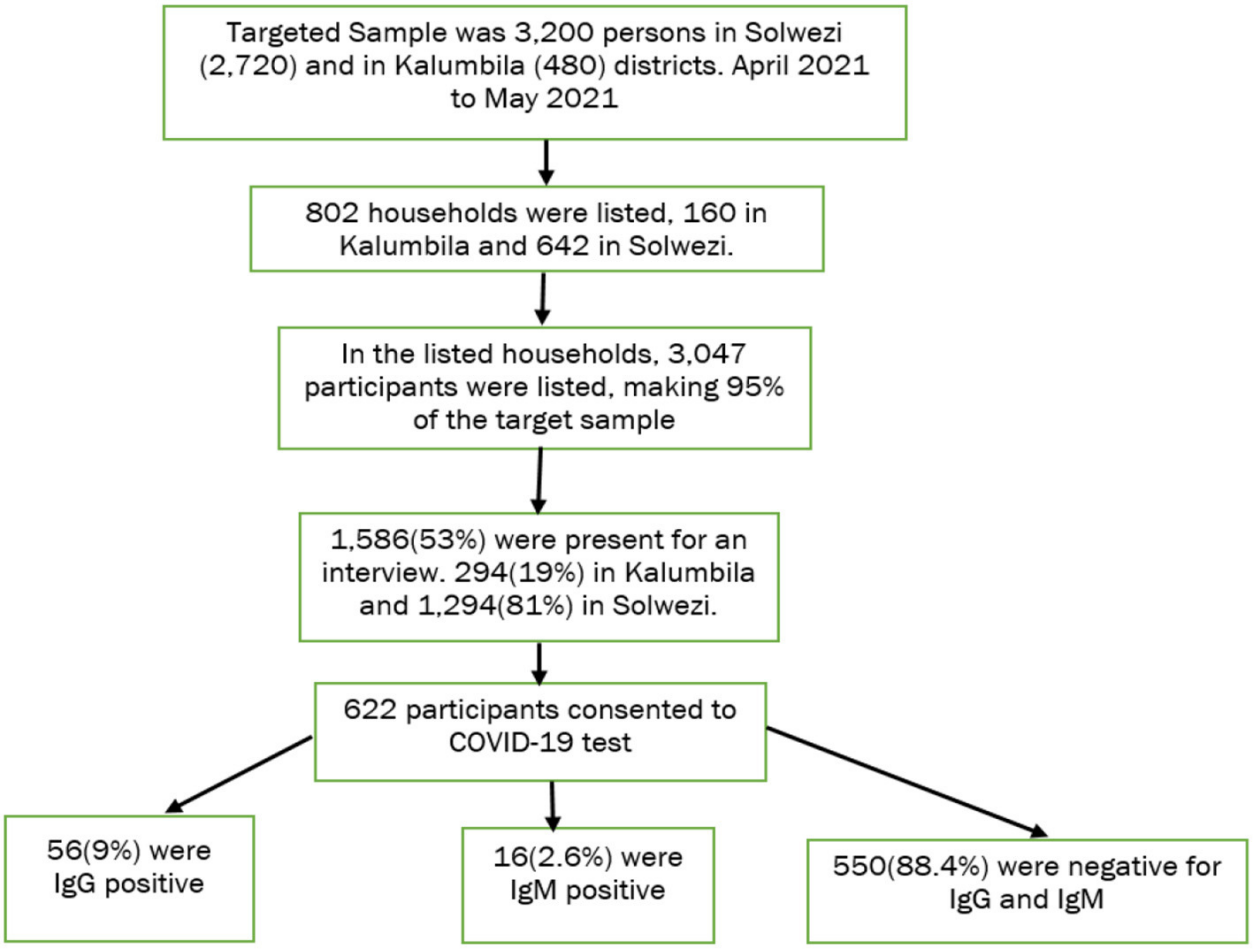
Flow chart of the sampling process and enumerated participants.

The targeted sample for the survey was 3,200 persons in Solwezi (2,720) and Kalumbila (480) districts. During the listing of households in the selected enumeration areas, 802 households were successfully listed out of the target of 800 households, that is, 160 in Kalumbila and 642 in Solwezi (Kansanshi). In the listed households, 3,047 persons were successfully listed, which made 95% of the target sample. However, during the actual survey, only 53% (1, 586) of the persons listed were present for the interview, that is, 294 (19%) in Kalumbila and 1, 294 (81%) in Solwezi (Figure 1).

#### Demographic characteristics of the participants

Table 1 reveals that the median age of the respondents was 26 years (IQR, 6–48). More females than males responded to the interview. In terms of marital status, the majority of those interviewed were married/cohabiting (55.9%). A large number of respondents interviewed had secondary education (46.2%). At the household level, 37.4% of the household heads had not changed their employment status after the COVID-19 outbreak. However, 15.9% of the respondents had reduced hours of work, while 13.0% of the participates were working from home and 9.3% had lost their jobs.

**Table 1:** Demographic characteristics and vaccine acceptability.

	**Total**		**Solwezi**		**Kalumbila**	
**Individual level**	***N** =* **1,586**		***N** =* **1,292**		***N** =* **294**	
	***N***	**%**	***N***	**%**	***N***	**%**
**Sex**						
Male	694	43.8	559	43.3	135	45.9
Female	892	56.2	733	56.7	159	54.1
**Age (median)**	**26 years**	**SD (14.97)**	**27 years**	**SD (15.42)**	**25 years**	**SD (12.72)**
**Age**						
5–9 years	215	13.6	175	13.5	40	13.6
10–14 years	201	21.7	164	12.7	37	12.6
15–19 years	171	10.8	142	11.0	29	9.9
20–24 years	168	10.6	141	10.9	27	9.2
25–29 years	237	14.9	173	13.4	64	21.8
30–39 years	319	20.1	259	20.0	60	20.4
40–49 years	275	17.3	238	18.4	37	12.6
**Current marital status**						
Married/Cohabiting	637	55.9	509	39.4	128	60.7
Never married	364	31.9	297	23.0	67	31.8
Divorced/Separated	68	6.0	58	4.5	10	4.7
Widowed	71	6.2	65	5.0	6	2.8
**Highest level of education completed**						
Primary	88	19.1	78	20.2	14	14.7
Secondary	213	46.2	169	46.2	44	46.3
Higher than secondary	160	34.7	123	33.6	37	38.9
**Household level**						
**Mean household size (SD)**	**802**	**3.88 (2.15)**	**625**	**3.87 (2.00)**	**160**	**3.92 (2.68)**
**Head of HH employment status after COVID-19**	**(*****N** **=*** **784)**		**(*****N** **=*** **625)**		**(*****N** **=*** **159)**	
I am still going to my workplace for same number of hours	293	37.4	172	27.5	121	76.1
as before the pandemic	145	18.5	140	22.4	5	3.1
I am still going to my workplace but am working reduced hours	125	15.9	105	16.8	20	12.6
I am working from home	102	13.0	95	15.2	7	4.4
I lost my job	73	9.3	68	10.9	5	3.1
I had to quit my job because I need to take care of people	9	1.1	8	1.3	1	0.6
who depend on me (children, parents)	37	4.7	37	5.9	0	0.0
**Household financial security after COVID-19**	**(*****N** **=*** **784)**		**(*****N** **=*** **625)**		**(*****N** **=*** **159)**	
More secure	49	6.3	47	7.5	2	1.3
Less secure	584	74.5	493	78.9	91	57.2
About the same	151	19.3	85	13.6	66	41.5
**Acceptability of COVID-19 vaccine by household head**						
**Getting myself vaccinated for COVID-19 would be a good way to protect myself against infection**	**(*****N** **=*** **716)**		**(*****N** **=*** **578)**		**(*****N** **=*** **138)**	
Strongly disagree	120	16.8	84	14.5	36	26.1
Disagree	160	22.3	124	21.5	36	26.1
Neither agree or disagree	97	12.1	85	14.7	12	8.7
Agree	267	33.3	235	40.7	32	23.2
Strongly agree	72	10.1	50	8.7	22	15.9
**I would allow my family members to be vaccinated against COVID-19**	**(*****N** **=*** **716)**		**(*****N** **=*** **578)**		**(*****N** **=*** **138)**	
Strongly disagree	124	17.3	88	15.2	36	26.1
Disagree	156	21.8	129	22.3	27	19.6
Neither agree or disagree	119	16.6	94	16.3	25	18.1
Agree	248	34.6	218	37.7	30	21.7
Strongly agree	69	9.6	49	8.5	20	14.5
**When many people in this community vaccinate against COVID-19, we will be protected against COVID-19**	**(*****N** **=*** **716)**		**(*****N** **=*** **578)**		**(*****N** **=*** **138)**	
Strongly disagree	77	10.8	55	9.5	22	15.9
Disagree	145	20.3	114	19.7	31	22.5
Neither agree or disagree	141	19.7	120	20.8	21	15.2
Agree	286	39.9	245	42.4	41	29.7
Strongly agree	67	9.4	44	7.6	23	16.7

SD, Standard deviation; N, number.

On household financial security that is the monthly income level of the household after the COVID-19, 74.5% of households that participated in the survey were less financially secure. However, little more respondents in the Kalumbila district indicated that their financial position had not changed, and this percentage was higher than that indicated by Solwezi district participants.

On the acceptability of the COVID-19 vaccine, almost half of the household heads agreed to get the vaccine and to allow their family members to get the vaccine. These household heads agreed that if many people in their community get vaccinated against COVID-19, the communities will be protected against COVID-19. However, ~39.1% of the household heads did not accept to get the vaccine. This non-acceptance of the vaccine was more in the Kalumbila district than the Solwezi district.

### COVID-19 prevalence

Table 2 reveals the COVID-19 experience of the respondents. When asked whether the respondents had experienced any symptoms of COVID-19 from 1 March 2020 and in the last 2 weeks, most of them reported that they had not. However, only 26.0% of the respondents had ever tested for COVID-19 (i.e., 23.4% in the Solwezi district and 37.8% in the Kalumbila district).

**Table 2:** COVID-19 characteristics.

	**Total**		**Solwezi**		**Kalumbila**	
**Individual level**	***N*** = **1,586**		***N*** = **1,292**		***N*** = **294**	
	***N***	**%**	***N***	**%**	***N***	**%**
	**1,586**		**1,292**		**294**	
**COVID-19 symptoms**						
**Since March 1, 2020, have you or someone in your home experienced any symptoms of COVID-19?**						
No	1447	91.2	1167	90.3	280	95.2
Yes, someone in my home (not including you)	27	1.7	24	1.9	3	1.0
Yes, multiple people in my home (not including)	17	1.1	17	1.3	0	0.0
Yes, I experienced such symptoms	71	4.5	62	4.8	9	3.1
Yes, both I and someone in my home had symptoms	8	0.5	6	0.5	2	0.7
I'm not sure/Don't know	16	1.0	16	1.2	0	0.0
**In the past 2 weeks, have you or someone in your home experienced any symptoms of COVID-19?**						
No	132	95.0	118	94.4	14	100.0
Yes, I experienced such symptoms	4	2.9	4	3.2	0	0.0
I'm not sure/Don't know	3	2.1	3	2.4	0	0.0
**Testing experience for COVID-19**						
**Have you ever been tested for COVID-19?**						
No	1,173	74.0	990	76.6	183	62.2
Yes	413	26.0	302	23.4	111	37.8
**Consent to test during this survey**						
**RDT Results**						
**IgG (Immunoglobulin G)**	**(*****N** **=*** **622)**		**(*****N** **=*** **509)**		**(*****N** **=*** **113)**	
Positive	56	9.0	50	9.8	6	5.3
Negative	566	91.0	459	90.2	107	94.7
**IgM (Immunoglobulin M)**						
Positive	16	2.6	14	2.8	2	1.8
Negative	606	97.4	495	97.2	111	98.2

SD, Standard deviation; N, number.

A total of 622 respondents consented to take a test for COVID-19 in this study. Of the total that tested for COVID-19, 9.0% were found positive for IgG (past infection) and 2.6% were found positive for IgM (recent infection). More positive respondents were found in the Solwezi district compared to the Kalumbila district.

### COVID-19 knowledge and attitude of COVID-19

Table 3 highlights the knowledge levels of respondents to COVID-19 and how they assess their self-risk. A few of the respondents were able to indicate the symptoms of COVID-19, and these were limited to those that were commonly mentioned in Zambia. Some differences in knowledge levels of knowing symptoms were observed among the respondents in the Solwezi and Kalumbila districts.

**Table 3:** COVID-19 knowledge and attitudes of COVID-19.

	**Total**		**Solwezi**		**Kalumbila**	
**Individual level**	***N** =* **1,586**		***N** =* **1,292**		***N** =* **294**	
	***N***	**%**	***N***	**%**	***N***	**%**
**Which of the following do you think are symptoms of COVID-19? – Knowledge of COVID-19**						
**Sore throat**						
Yes	696	43.9	613	47.4	83	28.2
No	890	56.1	679	52.6	211	71.8
**Fever**						
Yes	935	59.0	795	61.5	140	47.6
No	651	41.0	497	38.5	154	52.6
**Cough**						
Yes	982	61.9	830	64.7	152	51.7
No	604	38.1	462	35.8	142	48.3
**Running nose**						
Yes	747	47.1	626	48.5	121	41.2
No	839	52.9	666	51.5	173	58.8
**Shortness of breath at rest**						
Yes	501	31.6	421	32.6	80	27.2
No	1,085	68.4	871	67.4	214	72.8
**Shortness of breath when moving**						
Yes	284	17.9	231	17.9	53	18.0
No	1,302	82.1	1,061	82.1	241	82.0
**Chills**						
Yes	214	13.5	210	16.3	4	1.4
No	1,372	86.5	1,082	83.7	290	98.6
**Fatigue**						
Yes	230	14.5	207	16.0	28	7.8
No	1,356	85.5	1,085	84.0	271	92.2
**General lack of energy or malaise**						
Yes	89	5.6	83	6.4	6	2.0
No	1,497	94.4	1,209	93.6	288	98.0
**Loss of appetite**						
Yes	89	5.4	80	6.2	6	2.0
No	1,500	94.6	1,212	93.8	288	98.0
**Discomfort, tightness, or pressure in the chest**						
Yes	105	6.6	83	6.4	22	7.5
No	1,481	93.4	1,209	93.6	272	92.5
**Vomiting**						
Yes	41	2.6	36	2.8	5	1.7
No	1,545	97.4	1,256	97.2	289	98.3
**Nausea**						
Yes	50	3.2	48	3.7	2	0.7
No	1,536	96.8	1,244	96.3	292	99.3
**Diarrhea**						
Yes	85	5.4	79	6.1	6	2.0
No	1,501	94.6	1,213	93.9	288	98.0
**Muscle aches**						
Yes	75	2.8	70	5.4	5	1.7
No	1,542	97.2	1,222	94.6	289	98.3
**Headaches**						
Yes	385	24.3	291	22.5	94	32.0
No	1,201	75.7	1,001	77.5	200	68.0
**Joint aches**						
Yes	44	2.8	41	3.2	3	1.0
No	1,542	97.2	1,251	96.8	291	99.0
**Seizures**						
Yes	12	0.8	12	0.9	0	0.0
No	1,574	99.0	1,280	99.1	294	100.0
**Dizziness**						
Yes	16	1.0	15	1.2	1	0.3
No	1,570	99.0	1,277	98.8	293	99.7
**Feeling like it was difficult to stay awake**						
Yes	9	0.6	9	0.7	0	0.0
No	1,577	99.4	1,283	99.3	294	100.0
**Loss of ability to smell**						
Yes	170	10.7	138	10.7	32	10.9
No	1,416	89.3	1,154	89.3	262	89.1
**Loss of ability to taste**						
Yes	171	10.8	140	10.8	31	10.5
No	1,415	89.2	1,152	89.3	263	89.5
**How is COVID-19 transmitted? – COVID-19 knowledge**						
Close contact with an infected person who has symptoms	1,073	92.6	874	92.2	199	94.3
Close contact with an infected person even if they are not showing symptoms of infection	50	4.3	45	4.7	5	2.4
Contact with surfaces an infected person has touched	9	0.8	8	0.8	1	0.5
No knowledge of how its transmitted	27	2.3	21	2.2	6	2.8
**How likely do you think that the following events will happen in light of the current COVID-19?**						
**Self-assessed risk of getting COVID-19 – attitude**						
No chance	321	27.4	261	27.4	60	27.6
Very small chance	223	19.1	179	18.8	44	20.3
Medium chance	317	27.1	257	27.0	60	27.6
High chance	175	15.0	142	14.9	33	15.2
Very high chance	93	7.9	77	8.1	16	7.4
Absolutely sure	39	3.3	35	3.7	4	1.8
This has already happened	2	0.2	2	0.2	0	0.0

SD, Standard deviation; N, number.

Otherwise, many of them stated that they did not know the symptoms of COVID-19. When the participants were asked how one could get COVID-19, the majority felt it was through coming into contact with a COVID-19 patient with symptoms. However, only 2.3% of the respondents had no knowledge of how COVID-19 was transmitted. There were no observed differences between Solwezi and Kalumbila districts.

When the respondents were asked to gauge their risk of getting COVID-19, most of them assessed their self-risk to no chance (27.4%), very small chance (19.1%), and medium chance (27.1%). Even for this variable, the differences between Solwezi and Kalumbila districts were small.

### Trust information sources and prevention methods

Respondents were asked to gauge their trusted sources of information for COVID-19, as shown in Table 4. Most respondents indicated that they trusted information of COVID-19 from Doctors or other healthcare providers, with 56.2% of them responding that they completely trusted this source. Other sources of information completely trusted by respondents included the official Ministry of Health website (47.3%), the Press conference by the Minister of Health (46.8%), the Provincial or District Health Offices (46.5%), the World Health Organization (44.0%), the local media (42.1%), and the CDC (36.3%).

**Table 4:** COVID-19 trusted sources for COVID-19 information and effectiveness of prevention measures.

	**Total**		**Solwezi**		**Kalumbila**	
**Individual level**	***N** =* **1,170**		***N** =* **953**		***N** =* **217**	
	***N***	**%**	***N***	**%**	***N***	**%**
**How much do you trust the following sources to provide accurate COVID-19 information?**						
**Church**						
Not at all	209	17.9	165	17.3	44	20.3
Somewhat	289	24.7	237	24.9	52	24.0
Mostly	325	27.8	269	28.2	56	25.0
Completely	333	28.5	270	28.3	63	29.0
Not applicable	14	1.2	12	1.3	2	0.9
**Social media**						
Not at all	167	14.3	147	15.4	20	9.2
Somewhat	330	28.2	272	28.5	58	26.7
Mostly	350	29.9	275	28.9	75	34.6
Completely	233	19.9	186	19.5	47	21.7
Not applicable	90	7.7	73	7.7	17	7.8
**Newspaper**						
Not at all	159	13.6	143	15.0	16	2.8
Somewhat	251	21.6	187	19.6	64	29.5
Mostly	293	25.0	253	26.5	40	18.4
Completely	288	24.6	235	24.7	53	24.4
Not applicable	179	15.3	135	14.2	44	20.3
**Friends or family members**						
Not at all	79	6.8	73	7.7	6	2.8
Somewhat	348	29.7	271	28.4	77	35.5
Mostly	402	34.4	344	36.1	58	26.7
Completely	311	26.6	244	25.6	67	30.9
Not applicable	30	2.6	21	2.2	9	4.1
**Coworkers or classmates**						
Not at all	124	10.6	115	12.1	9	4.1
Somewhat	320	27.4	255	26.8	65	30.0
Mostly	306	26.2	247	25.9	59	27.2
Completely	232	19.8	181	19.0	51	23.5
Not applicable	188	16.1	155	16.3	33	15.3
**Doctors or other healthcare provider**						
Not at all	39	3.3	36	3.8	3	1.4
Somewhat	95	8.1	86	9.0	9	4.1
Mostly	352	30.1	308	32.3	44	20.3
Completely	657	56.2	502	52.7	155	71.4
Not applicable	27	2.3	21	2.2	6	2.8
**Official ministry of health website**						
Not at all	143	12.2	135	14.2	8	3.7
Somewhat	88	7.5	59	6.2	29	13.4
Mostly	247	21.1	219	23.0	28	12.9
Completely	553	47.3	426	44.7	127	58.5
Not applicable	139	11.9	114	12.0	25	11.5
**Official ZNPHI website**						
Not at all	171	14.6	154	16.2	17	7.8
Somewhat	95	8.1	70	7.3	25	11.5
Mostly	238	20.3	204	21.4	34	15.7
Completely	492	42.1	385	40.4	107	49.3
Not applicable	174	14.9	140	14.7	34	15.7
**DMMU official website**						
Not at all	179	15.3	160	16.8	19	8.8
Somewhat	136	11.6	106	11.1	30	13.8
Mostly	240	20.5	208	21.8	32	14.7
Completely	385	24.3	289	30.3	96	44.2
Not applicable	230	14.5	190	19.9	40	18.4
**Press conference by the minister of health**						
Not at all	130	11.1	117	12.3	13	6.0
Somewhat	91	7.8	62	6.5	29	13.4
Mostly	260	22.2	221	23.2	39	18.0
Completely	547	46.8	432	45.3	115	53.0
Not applicable	142	12.1	121	12.7	21	9.7
**World health organization (WHO)**						
Not at all	158	13.5	138	14.5	20	9.2
Somewhat	86	7.4	66	6.9	20	9.2
Mostly	231	19.7	204	21.4	27	12.4
Completely	515	44.0	393	41.2	122	56.2
Not applicable	180	15.4	152	15.9	28	12.9
**Centers for disease control and prevention (CDC)**						
Not at all	168	14.4	144	15.1	24	11.1
Somewhat	109	9.3	80	8.4	29	13.4
Mostly	248	21.2	222	23.3	26	12.0
Completely	425	36.3	320	33.6	105	48.4
Not applicable	220	18.8	187	19.6	33	11.2
**Provincial or district health office**						
Not at all	140	12.0	128	13.4	12	5.5
Somewhat	96	8.2	63	6.6	33	15.2
Mostly	276	23.6	232	24.3	44	20.3
Completely	544	46.5	428	44.9	116	53.3
Not applicable	114	9.7	102	10.7	12	5.5
**Media (i.e., ZNBC)**						
Not at all	69	5.9	60	6.3	9	4.1
Somewhat	128	10.9	97	10.2	31	14.3
Mostly	422	36.1	355	37.3	67	30.9
Completely	493	42.1	394	41.3	99	45.6
Not applicable	58	5.0	47	4.9	11	5.1
**Community leaders**						
Not at all	183	15.6	128	13.4	55	25.3
Somewhat	228	19.5	169	17.7	59	27.2
Mostly	315	26.9	275	28.9	40	18.2
Completely	279	23.8	220	23.1	59	27.2
Not applicable	165	14.1	161	16.9	4	1.8
**Given the state of the COVID-19 pandemic today and the associated spread, how effective do you think the following policy measures are?**						
**Close schools and daycares**						
Not effective at all	388	33.2	305	32.0	83	38.2
Hardly effective	173	14.8	115	12.1	58	26.7
Somewhat effective	220	18.8	191	20.0	29	13.4
Effective	289	24.7	270	28.3	19	8.8
Very effective	100	8.5	72	7.6	28	12.9
**Close bars**						
Not effective at all	159	13.6	121	12.7	38	17.5
Hardly effective	86	7.4	62	6.5	24	11.1
Somewhat effective	167	14.3	150	15.7	17	7.8
Effective	485	41.5	446	46.8	39	18.0
Very effective	273	23.3	174	18.3	99	33.7
**Close restaurants**						
Not effective at all	209	17.9	166	17.4	43	19.8
Hardly effective	143	12.2	105	11.0	38	17.5
Somewhat effective	296	25.3	233	24.4	63	29.0
Effective	407	34.8	378	39.7	29	13.4
Very effective	115	9.8	71	7.5	44	20.3
**Do not allow visitors in hospital**						
Not effective at all	148	12.6	104	10.9	44	20.3
Hardly effective	145	12.4	86	9.0	59	27.3
Somewhat effective	273	23.3	240	25.2	33	15.2
Effective	495	42.3	459	48.2	36	16.6
Very effective	109	9.3	64	6.7	45	20.7
**Disinfection of public places**						
Not effective at all	66	5.6	51	5.4	15	6.9
Hardly effective	76	6.5	58	6.1	18	8.3
Somewhat effective	167	14.3	142	14.9	25	11.5
Effective	627	53.6	561	58.9	66	30.4
Very effective	234	20.0	141	14.8	93	42.9
**Universal wearing of face masks**						
Not effective at all	46	3.9	40	4.2	6	2.8
Hardly effective	63	5.4	54	5.7	9	4.1
Somewhat effective	112	9.6	103	10.8	9	4.1
Effective	534	45.6	488	51.2	46	21.2
Very effective	415	35.5	268	28.1	147	67.7

SD, Standard deviation; N, number.

Furthermore, the respondents were asked how effective the policy measures were to control COVID-19 spread. Most respondents stated that disinfection of public places (53.6%), universal wearing of face masks (45.6%), not allowing visitors in hospitals (42.3%), closing bars (41.5%), and closing restaurants (34.8%) were indicated as the most effective measures of controlling COVID-19.

### Lifestyle or daily activities change during COVID-19

In Table 5, respondents were asked whether they have made lifestyle changes to prevent COVID-19 infection, and the majority of them indicated that they are practicing social distancing, staying at home more, hand washing more than usual, hand sanitizing more than usual, and wearing face masks when out in public. However, some respondents indicated that they are not adhering to the following: cleaning their houses more than usual, disinfecting surfaces in their households more than usual, avoiding or canceling domestics travel, avoiding or canceling international travel, eating from restaurants, and drinking from bars or nightclubs.

**Table 5:** Lifestyle or daily activities change during COVID-19.

	**Total**		**Solwezi**		**Kalumbila**	
**Individual level**	***N** =* **1,586**		***N** =* **953**		***N** =* **294**	
	***N***	**%**	***N***	**%**	***N***	**%**
**Have you made any of the following changes to your lifestyle or daily activities because of COVID-19?**						
**Have you practiced distancing**						
Yes	1,094	93.5	892	93.6	202	93.1
No	76	6.5	61	6.4	15	6.9
**Have you stayed at home as much as possible**						
Yes	1,058	90.4	883	92.7	175	80.6
No	112	9.6	70	7.3	42	19.4
**More handwashing than usual**						
Yes	1,066	67.2	877	67.9	189	64.3
No	520	32.8	415	32.1	105	46.9
**More use of hand sanitizer than usual**						
Yes	944	59.5	788	61.0	156	53.1
No	642	40.5	504	39.0	138	46.9
**More cleaning in your home than usual**						
Yes	660	41.6	578	44.7	82	27.9
No	926	58.4	714	55.3	212	72.1
**More disinfecting surfaces in your household than usual**						
Yes	380	24.0	340	26.3	40	13.6
No	1,206	76.0	952	73.7	254	86.4
**Avoiding or canceling domestic travel**						
Yes	259	16.3	191	14.8	68	23.1
No	1,327	83.7	1,101	85.2	226	76.9
**Avoiding or canceling international travel**						
Yes	141	8.9	112	8.7	29	9.9
No	1,445	91.1	1,180	91.3	265	90.1
**Not eating from restaurants**						
Yes	268	16.9	175	3.5	93	31.6
No	1,318	83.1	1,117	86.5	201	68.4
**Avoid drinking from bars or nightclubs**						
Yes	286	18.0	239	18.5	47	16.0
No	1,300	82.0	1,053	81.5	247	84.0
**Wearing a mask when out in public**						
Yes	880	55.5	700	54.2	180	61.2
No	705	44.5	592	45.8	114	38.8
**How much has the pandemic impacted your day-to-day life?**						
It has not impacted my life at all	103	8.8	58	6.1	45	20.7
It has impacted my life a little	294	25.1	252	26.4	42	19.4
It has moderately impacted my life	366	31.3	302	31.7	64	29.5
It has extremely impacted my life	402	34.4	336	35.3	66	30.4
Refused to answer	5	0.4	5	0.5	0	0.0

SD, Standard deviation; N, number.

When asked whether COVID-19 has had an impact on the respondent's day-to-day life, 34.4% of them indicated that COVID-19 has had an extreme impact on their life, whereas 8.8% of the respondents revealed that COVID-19 has had no impact on their lives.

#### Factors associated with COVID-19 infection

The chi-square test was done to determine associations between categorical variables and COVID-19 infection. Table 6 shows the results between participant socio-demographic characteristics and COVID-19 infections as determined by Pearson's chi-square test of independence. Female participants had higher COVID-19 positive results (8.2%) (49/594) than male participants (5.4%) (32/594). Nonetheless, there was no significant difference in COVID-19 positive test results of males and females (*p* = 0.110). The study further showed that participants who were in Solwezi tested positive for COVID-19 (49/594) (8.3%) compared to those that were in Kalumbila. There was a significant difference in the positivity of the test done in Kalumbila and those in Solwezi as evidenced by the p-value (*p* = 0.043). The results, however, showed no significant difference in those participants who have tested for COVID-19 before the survey was conducted and those who tested when the survey was conducted (*p* = 0.145).

**Table 6:** Cross-tabulation of the predictors of COVID-19 infection.

**Factor**	**COVID-19 results**		***p*-value**
	**Negative**, ***N*** = **545 (92.0%)**	**Positive**, ***N*** = **49 (0.8%)**	
**Age of participants**			
**6–14 years**	134 (96.4%)	5 (3.6)	
**14–24 years**	125 (92.6%)	10 (7.4%)	
**>25 years**	286 (89.4%)	34 (10.6%)	0.114^C, M^
**Sex**			
Male	294 (90.2%)	32 (9.8%)	
Female	251 (93.7%)	49 (6.3%)	0.110^C, M^
**Marital status**			
Married	192 (90.1%)	21 (9.9%)	
Unmarried	353 (92.7%)	28 (7.3%)	0.110^C, M^
**Education level**			
Primary	172 (90.5%)	11 (9.5%)	
Secondary	198 (93.8%)	13 (6.2%)	
College/ University	175 (90.7%)	18 (9.3%)	0.577^C, M^
**District**			
Kalumbila	104 (96.3%)	4 (3.7%)	
Solwezi	545 (91.7%)	49 (8.3%)	0.043^C, M^
**Ever tested for COVID-19**			
No	543 (91.9%)	48 (8.1%)	
Yes	2 (6.7%)	1 (3.3%)	0.145^C^
**Experienced any COVID-19 symptoms**			
No	469 (93.4%)	33 (6.6%)	
Yes	76 (82.6)	16 (17.4.5)	0.005^C, M^
**Allow family members to get vaccine**			
Disagree	220 (90.1%)	22 (9.1%)	
Neutral (Neither disagree nor Agree)	86 (93.5%)	6 (6.5%)	
Agree	239 (91.9%)	21 (8.1%)	0.633^C, M^
**Vaccine protect community**			
Disagree	164 (92.1%)	14 (7.9%)	
Neutral (Neither disagree nor Agree)	124 (93.9%)	8 (6.1%)	
Agree	545 (91.8%)	49 (8.2%)	0.404 ^C, M^
**Vaccine will protect individual**			
Disagree	215 (90.7%)	22 (92.8%)	
Neutral	79 (94.1%)	4 (6.0%)	
Agree	251 (91.9%)	22 (8.1%)	0.481^C, M^

^C^Chi-squared test of association. ^M^showing that there were missing values but p-values were obtained on complete case analysis.

There was no evidence of a difference in testing positive for COVID-19 between age groups, whether one was married or not, whether attained a certain level of education or not, whether the participant will allow a family member to have a vaccine or not, whether the participants disagreed or agreed that COVID-19 vaccine will protect the community, and whether the participants will disagree or agree that COVID-19 vaccine will protect individuals. However, there was a significant difference in who did not experience COVID-19 compared to those that did experience as evidenced by the p-value (*p* = 0.006).

#### Predictors of the prevalence of COVID-19 infection among the participants

A logistic regression was performed to examine the predictors of the prevalence of COVID-19 participants of 5 years and above. The significance level was set at a *p* < 0.05 at 95% confidence interval.

The results of univariate analysis, that is, crude odds ratios (cOR) in Table 7, show that participants in Kalumbila, that is, participants who lived in Solwezi mining community had 2.7 times the odds of testing positive for COVID-19 compared to participants who lived in Kalumbila mining community (cOR = 2.65; 95% CI: 1.00, 7.01), participants whose age, that is, age above 24 years had 3.2 times the odds of being testing positive for COVID-19 compared to participants who age was between 6 and 14 years (cOR = 3.19, 95% CI: 1.01, 10.05), and also participants who had or their family member had experienced COVID-19 symptoms before had 3.0 times the odds of testing positive for COVID-19 compared to those who never experienced COVID-19 symptoms before the study was done (cOR = 2.99, 95% CI: 1.50, 5.96), and participants marital status (cOR = 0.73; 95% CI: 0.30, 1.77), sex of participant (cOR = 0.62; 95% CI: 0.33, 1.15) and educational level, secondary (cOR = 0.63; 95% CI: 0.20, 2.07) university/college (cOR = 0.98; 95% CI: 0.42, 2.28) compared to primary level had no statistically significant association with testing positive for COVID-19.

**Table 7:** Logistic regression adjusted and unadjusted.

	**Unadjusted**	***p*-value**	**Adjusted**	***p*-value**
	**Odd ratios (95% CI)**		**Odds ratios (95%CI)**	
**Age, years**				
5–14	Ref (1)		Ref (1)	
15–24	2.14 (0.55, 8.40)	0.234	1.50 (0.47, 4.75)	0.443
>24	3.19 (1.01, 10.05)	0.048	2.72 (0.81, 9.08)	0.092
**Gender**				
Male	Ref (1)		Ref (1)	
Female	0.62 (0.33, 1.15)	0.112	0.68 (0.38, 1.19)	0.153
**Marital status**				
Unmarried	Ref (1)		Ref (1)	
Married	0.73 (0.30, 1.77)	0.431	0.75 (0.35, 1.61)	0.41
**Education level**				
Primary	Ref (1)		Ref (1)	
Secondary	0.63 (0.20, 2.07)	0.394	0.66 (0.19, 2.25)	0.454
University/College	0.98 (0.42, 2.28)	0.963	0.91 (0.36, 2.28)	0.814
**Test for COVID-19**				
No	Ref (1)		Ref (1)	
Yes	5.66 (0.35, 92.3)	0.19	5.67 (0.19, 168.27)	0.272
**Experience COVID-19 symptoms**				
No	Ref (1)		Ref (1)	
Yes	2.99 (1.50, 5.96)	0.006	2.63 (1.31, 5.27)	0.012
**Household members**				
4 January	Ref (1)		Ref (1)	
9 May	1.15 (0.52, 2.54)	0.694	1.02 (0.48, 2.15)	0.948
12 October	1.96 (0.14, 26.88)	0.569	1.70 (0.21, 13.55)	0.569
**Districts**				
Kalumbila	Ref (1)	Ref	Ref (1)	
Solwezi	2.65 (1.00, 7.01)	0.049	2.28 (0.69, 7.53)	0.151
**Family member getting vaccinated**				
Disagree	Ref (1)		Ref (1)	
Neither	1.70 (0.35, 1.40)	0.264	1.26 (0.47, 3.35)	0.605
Agree	0.88 (0.41, 1.86)	0.702	0.79 (0.11, 5.81)	0.792
**Vaccine will protect community**				
Disagree	Ref (1)		Ref (1)	
Neither disagree/agree	0.76 (0.30, 1.90)	0.503	1.15 (0.40, 3.26)	0.762
Agree	1.23 (0.60, 2.52)	0.523	2.66 (0.61, 11.74)	0.165
**Vaccine will protect me**				
Disagree	Ref (1)		Ref (1)	
Neither disagree/agree	0.62 (0.25, 1.53)	0.255	0.54 (0.14, 2.13)	0.335
Agree	0.85 (0.46, 1.58)	0.578	0.46 (0.15, 1.46)	0.161

On the other hand, participants who had a COVID-19 test before the study (cOR = 5.66; 95% CI: 0.35, 92.3), household members 5 to 9 (cOR = 1.15; 95% CI: 0.52, 2.54), household members 10 to 12 (cOR = 1.96; 95% CI: 0.14, 26.88) compared to those with 1–4 household members, whether the household members would be allowed to receive the vaccine by household head, neither disagree nor agree (cOR = 1.70; 95% CI: 0.35, 1.40), agree (cOR = 0.88; 95% CI: 0.41, 1.86), will you accept the vaccine as an individual, neither or agree (cOR = 0.62; 95% CI: 0.25, 1.53), agree (cOR = 0.85; 95% CI: 0.46, 1.58), and will the vaccine protect the community, neither disagree nor agree (cOR = 0.76; 95% CI: 0.30, 1.90), agree (cOR = 1.23; 95% CI: 0.60, 2.52) had no statistically significant association with testing positive for COVID-19.

Table 8 shows the final multivariable analysis model arrived that fit the data well. Multiple regression was done in order to control for possible confounding. An investigator-led stepwise regression was used to arrive at the model. This implies running the multiple logistic regression command with all the predictor variables in the first stage and then removing variables with the highest *p*-values one by one from the model until we remained with a model that best explained the data (parsimonious model). The multivariable analysis model contains six explanatory variables: participants' age, sex, whether the participant tested for COVID-19 before the study or not, whether the participants experienced symptoms of COVID-19 or not, whether participants agreed, disagreed, or neither disagreed nor agreed that vaccinating the community would protect them from COVID-19, and whether an individual getting the vaccine would protect them from COVID-19 as the best predictors of being found COVID-19 positive. Although participants sex, whether the participant tested for COVID-19 before the study or did not, whether participants agreed, disagreed, or neither disagreed nor agreed that vaccinating the community would protect them from COVID-19, and whether an individual getting the vaccine would protect them from COVID-19 were not statistically significant, the variables were left in the model due to prior knowledge from other studies which consistently showed that they could be used to perfectly predict one being found positive.

**Table 8:** Multivariable analysis.

**Testing positive**	**Odds ratios**	**95% confidence intervals**	***p*-values**
**Age, years**			
5–14	Ref	−1	
15–24	1.75	(0.57, 5.36)	0.328
>24	2.94	(1.10, 7.81)	0.031
**Sex**			
Male	Ref	−1	
Female	0.65	(0.34, 1.22)	0.181
**Tested for COVID-19**			
No	Ref	−1	
Yes	5.77	(0.43, 77.73)	0.187
**Experience COVID-19 symptoms**			
No	Ref	−1	
Yes	2.6	(1.33, 5.05)	0.005
**Vaccine will protect community**			
Disagree	Ref	−1	
Neither disagree/agree	1.26	0.43, 3.72	0.676
Agree	2.36	0.86, 6.49	0.095
**Vaccine will protect me**			
Disagree	Ref	−1	
Neither disagree/agree	0.66	(0.20, 2.15)	0.488
Agree	0.41	(0.16, 1.06)	0.065

As shown in Table 8, age and having experienced symptoms of COVID-19 before the study were significantly associated with being found positive for COVID-19. The effect of experiencing COVID−19 symptoms, that is, whether the participant had experienced symptoms before the study, was 2.6 times more likely to be infected (AOR = 2.60; 95% CI: 1.33, 5.05) compared to participants who did not have any COVID-19 symptoms before the study, holding constant the effect of other predictors in the model. The other predictor was age, participants with age >24 years were 1.5 times more likely to be positive for COVID−19 (AOR =2.94; 95% CI = 1.10, 7.81) compared to other age categories holding constant the effects of other predictors in the model. However, sex (AOR = 0.65; 95% CI: 0.34, 1.22), and whether they tested for COVID-19 before the study or not (AOR = 5.77; 95% CI: 0.43, 77.73), whether the vaccine will protect the community, neither disagree nor agree (AOR = 1.26; 95% CI: 0.43, 3.72), agree (AOR = 2.36; 95% CI: 0.86, 6.49) compared to those who disagree. Whether the vaccine will protect the individual, neither disagree nor agree (AOR = 0.66; 95% CI: 0.20, 2.15), agree (AOR = 0.41; 95% CI: 0.16, 1.06) compared to participants who disagreed had no statistically significant association with healthcare-seeking behaviors controlling for the effect of other predictors in the model.

## Discussion

The study aimed at understanding disease transmission in its wider community in Solwezi and Kalumbila to determine the effectiveness of controls and the demographic spread of COVID-19. The results have shown that the prevalence of COVID-19 in the two mining towns of Solwezi and Kalumbila was 9.0% for previous infections and 2.6% for active cases. The findings are not different from what was reported in six districts of Zambia, which showed that 5–11% of the population was infected, while previous infections were ~8.2% (13). In contrast, a study done in Jakarta (24) found a much higher prevalence of 15.7% compared to our finding, which was the same period this survey was conducted, with the highest infection rates occurring in March 2020 (26.3%), followed by January 2021 (23.9%) and February 2021 (21.8%).

The study has shown that older age groups were more likely to be found with SARS-CoV-2. This is not a different study done in Zambia that found that age youngest age group, 15 to 19 years, had the lowest seroprevalence of SARS-CoV-2 (25). The findings have been demonstrated elsewhere that combined measure's pooled SARS-CoV-2 prevalence rose with age (13). Another study found that the only age groups where SARS-CoV-2 transmission has persisted with reproduction numbers (transmission rates) continuously over one are people aged 20 and above (26).

Furthermore, the study has shown that the results have also revealed that the knowledge of COVID-19 was limited to the general public provided during sensitization in most of the communities and hence increasing infections. It has been demonstrated elsewhere (27) that greater awareness and attitude toward COVID-19 prevention methods can reduce transmission rates and enhance public health results. This is in line with the findings of another study (28) that found that little was known about the general Zambian population's views and awareness about COVID-19 preventative efforts.

This study has further established that strong preventative and control measures undertaken by local governments such as the use of mask and hand washing in every public area are mostly responsible for reductions in infections among community members and the prevalence of COVID-19 was still high. A study done in Zambia (28) found that despite the awareness of COVID-19 preventative measures being high, people's opinions about these measures were unfavorable and may have an impact on people adherence to COVID-19 measures. Another study argues that adherence to COVID-19 preventive measures was correlated with occupation and knowledge (29). With the problem of adherence to public health measures because of false information which has presented difficulties in Zambia's fight against COVID-19, continuous public education and sensitization on COVID-19 and the value of vaccinations are required. Vaccines boost immune function and help the body fight against infectious invaders (30). Vaccines are highly successful at containing disease outbreaks, according to the available data (31). Similar research has demonstrated the high efficacy and safety of COVID-19 vaccinations (31–33).

The study has demonstrated that most of the information that is trusted about COVID-19 is from the health providers and generally from the Ministry of Health as well as WHO and Center for Disease Control (CDC). The findings in this study are in line with the findings from other studies (34), which observed that willingness to participate in contact tracing was positively correlated with trust in information from public health organizations, even though these organizations were not always the primary providers of information regarding COVID-19. In contrast, a study (35) found that when it comes to knowledge regarding COVID-19 vaccinations, doctors have more faith in official government sources and their employers than do nurses, pharmacists, and advanced practice providers (35).

Furthermore, the study demonstrated that COVID-19 had a negative impact on the two communities in terms of financial insecurity (monthly income) and job losses. According to a study (36), it was found that 24% of respondents lost their jobs or income and had reduced hours of work due to the COVID-19 pandemic. In contrast with our findings, another study (37) found that the need for specialized skills in the logistics and healthcare industries was expanded. Furthermore, there is evidence of a rise in job openings incorporating remote working conditions (37).

The study further demonstrates that vaccine acceptability is mixed with some agreeing to taking the vaccine and recommending it to their family members and other persons in their communities, whereas other persons totally refused to accept the COVID-19 vaccine. This is in line with what has been reported elsewhere (38) that the acceptance rate of the COVID-19 vaccination was just 37.4% due to hesitance. In contrast to our finding, a study done in Japan (39) found that participants who had received the seasonal influenza vaccination had higher acceptance rates for the COVID-19 vaccine. Another study found that participants were more inclined to take the COVID-19 shots because they thought that vaccines are typically safe and if they were prepared to pay for them once they became available (38). However, in line with this study finding, participants who were working and older than 35 years were less likely to receive the COVID-19 vaccinations (40). Additionally, individuals who distrusted all sources of information about COVID-19 vaccinations and those who thought there was a conspiracy behind the disease were less likely to accept the vaccine (38).

### Strength of the study

The study has highlighted gaps, such as a lack of adherence to COVID-19 preventive measures, as a result of a lack of knowledge and misinformation on vaccine uptake. Therefore, this is a good opportunity for the Ministry of Health and other partners in Zambia to work together to put up the right message and to sensitize the community to the importance of the vaccine as it stimulates the immune system and aids in the fight against infectious agents. A random sample of the population was used in our study. This study further highlights the mixed feeling about the vaccine uptake from participants in the mining communities. It has also demonstrated that it is important to strengthen the level of knowledge about COVID-19 among community members by emphasizing good practices as it can help reduce the disease burden among workers who work for the mining community. Very few studies have been done with a focus on the prevalence of COVID-19 in mining communities.

### Limitations of the study

This study is not free from limitations mainly resulting from being a cross-sectional study, and it can only provide information at one point in time. As such, it is not possible to draw firm conclusions on the evolution of the pandemic in the two communities. Despite the robust sampling process that was used to select SEA and households, the results reported in this study are limited to the two mining communities. The target household size estimated for the study was four persons. During the listing of households, the number of persons per household was almost the target size. However, during the actual survey, the number of household members available per household was less. The less members per household could have biased the findings of the study, especially if those that were not available had different attributes from those present.

## Conclusion

The prevalence of COVID-19 in the mining towns for previous infections and active cases was similar to previous studies done in Zambia. Older age groups and being symptomatic were associated with COVID-19 infection. The study has shown that the knowledge of COVID-19 was limited to the general public provided during sensitization in the mining communities, leading to increased infections. Greater awareness and attitudes toward COVID-19 prevention methods can reduce transmission rates and enhance public health results. Local governments' strong preventative and control measures, such as mask use and hand washing in public areas, could reduce infections among community members. Many community members were probably asymptomatic and, as such, gauged themselves as having a low risk of getting infected with COVID-19. This resulted in most community members not adhering to public health preventive measures. The government and partners should continue to sensitize the community members on the preventive measures of COVID-19 in spite of not being adhere to and continue with community testing so that all those positive but without symptoms can self-isolate and those with symptoms and who are sick can be admitted to the hospital. There should also be a campaign in promoting vaccine uptake among community members.

## Data availability statement

The raw data supporting the conclusions of this article will be made available by the authors, without undue reservation.

## Ethics statement

The studies involving humans were approved by University of Zambia, Biomedical Research Ethics Committee. The studies were conducted in accordance with the local legislation and institutional requirements. Written informed consent for participation in this study was provided by the participants' legal guardians/next of kin.

## Author contributions

Conceived, composed the manuscript, the manuscript was rewritten, updated, and improved by TM, KMe, NK, MD, and KMw. The essay was co-written by all the authors, who also gave their approval to the final draft. All authors contributed to the article and approved the submitted version.
